# An interaction between myosin-10 and the cell cycle regulator Wee1 links spindle dynamics to mitotic progression in epithelia

**DOI:** 10.1083/jcb.201708072

**Published:** 2018-03-05

**Authors:** Joshua C. Sandquist, Matthew E. Larson, Sarah Woolner, Zhiwei Ding, William M. Bement

**Affiliations:** 1 Biology Department, Grinnell College, Grinnell, IA; 2 Department of Zoology, University of Wisconsin-Madison, Madison, WI; 3 Cellular and Molecular Biology Graduate Program, University of Wisconsin-Madison, Madison, WI; 4 Medical Scientist Training Program, University of Wisconsin-Madison, Madison, WI; 5 Laboratory of Cell and Molecular Biology, University of Wisconsin-Madison, Madison, WI; 6 Wellcome Trust Centre for Cell-Matrix Research, Division of Developmental Biology and Medicine, School of Medical Sciences, Faculty of Biology, Medicine and Health, Manchester Academic Health Science Centre, University of Manchester, Manchester, England, UK

## Abstract

Anaphase in epithelia typically does not ensue until after spindles have achieved a characteristic position and orientation, but how or even if cells link spindle position to anaphase onset is unknown. Here, we show that myosin-10 (Myo10), a motor protein involved in epithelial spindle dynamics, binds to Wee1, a conserved regulator of cyclin-dependent kinase 1 (Cdk1). Wee1 inhibition accelerates progression through metaphase and disrupts normal spindle dynamics, whereas perturbing Myo10 function delays anaphase onset in a Wee1-dependent manner. Moreover, Myo10 perturbation increases Wee1-mediated inhibitory phosphorylation on Cdk1, which, unexpectedly, concentrates at cell–cell junctions. Based on these and other results, we propose a model in which the Myo10–Wee1 interaction coordinates attainment of spindle position and orientation with anaphase onset.

## Abstract (precis)

Proper spindle orientation must be achieved before anaphase onset, but whether and how cells link spindle position to anaphase onset is unknown. Sandquist, Larson, et al. identify a novel interaction between the motor protein myosin-10 and the cell cycle regulator wee1 that is proposed to help coordinate preanaphase spindle dynamics and positioning with mitotic exit.

## Introduction

Mitotic spindles in epithelia typically attain a characteristic position and orientation before anaphase (Baena-López et al., 2005; Lechler and Fuchs, 2005; da Silva and Vincent, 2007; Mao et al., 2011). In the most common example, “symmetric” division, the spindle is positioned in the approximate middle of the x–y plane and is oriented parallel to the epithelial layer (Gillies and Cabernard, 2011; Morin and Bellaïche, 2011; Bergstralh et al., 2017). This ensures that cytokinesis, which divides the cell between the separating chromosomes, maintains epithelial architecture by directing formation of two equal-sized daughter cells in the plane of the epithelium.

It is now clear that the spindle achieves its final position and orientation during symmetric division via a combination of cytoskeletal motor-dependent movement and cortical anchoring complexes (Woolner and Papalopulu, 2012; Kiyomitsu and Cheeseman, 2013; di Pietro et al., 2016). It is also clear that failure of proper symmetric positioning results in a variety of pathological consequences, including disrupted tissue architecture and promotion of metastasis (Vasiliev et al., 2004; Fish et al., 2006; Quyn et al., 2010). What remains unclear is whether or how epithelial cells link spindle position to cell cycle progression. In principle, such a mechanism may be unnecessary if the achievement of metaphase takes longer than spindle positioning and if both occur simultaneously. However, in a variety of intact epithelia, spindle positioning and orientation do not commence until after metaphase and, further, the interval from metaphase to anaphase can be many minutes (Adams, 1996; Haydar et al., 2003; Woolner et al., 2008; Peyre et al., 2011; Larson and Bement, 2017), suggesting that epithelial cells can delay anaphase until the spindle has achieved the correct position and orientation.

Consistent with this hypothesis, automated analysis of mitotic dynamics in 100 *Xenopus* embryonic epithelial cells revealed that spindles execute a stereotyped, two-part “dance” after achieving metaphase. First, spindles undergo a slow rotational movement until they are parallel to the long axis of the cell; second, they undergo rapid oscillatory movements to and from the cortex, which culminate in x–y plane centering (Larson and Bement, 2017). Strikingly, anaphase onset is temporally correlated with “on target” cortical contacts by the spindle poles (i.e., contact with cortical positions on the same axis as that defined by the final orientation of the spindle). Based on these results, it was proposed that the spindle dance is part of a mechanism that epithelial cells use to link mitotic progression to proper spindle positioning and orientation.

Myosin-10 (Myo10), a microtubule-binding, actin-based motor protein that has been previously implicated in spindle dynamics and mitotic progression in *Xenopus laevis* embryonic epithelia, is a strong candidate contributor to the mechanism suggested above (see previous paragraph). Depletion of Myo10 results in spindle lengthening, pole fragmentation, and metaphase delay (i.e., an increase in the amount of time the cells spend between metaphase and anaphase; Woolner et al., 2008), whereas dominant-negative expression of the isolated Myo10 MyTH4 domain, which mediates Myo10’s interaction with microtubules (Hirano et al., 2011), produces only some of these phenotypes. Specifically, whereas a high level MyTH4 expression results in pole fragmentation and a metaphase delay, moderate expression produces only the delay (Sandquist et al., 2016), indicating that this fragment produces more limited phenotypes than Myo10 depletion by competing with endogenous Myo10 for binding to some unidentified target. This target is not, apparently, microtubules in that expression of the MyTH4-DD mutant, which is deficient in microtubule binding (Hirano et al., 2011), is at least as efficient in causing metaphase delay as wild-type MyTH4 and is apparently more specific in so doing because it does not result in spindle pole fragmentation, even at higher expression levels (Sandquist et al., 2016). Here we identify Wee1, a cell cycle regulatory kinase, as a Myo10-binding partner and explore the possibility that this interaction is part of a mechanism linking spindle dynamics and positioning to mitotic progression.

## Results and discussion

### Myo10–Wee1 interaction

The MyTH4 domain of Myo10 makes up half of the so-called MyTH4-FERM (4.1 and ezrin/radixin/moesin) cassette, which is present in several myosins and mediates binding with multiple proteins (Zhang et al., 2004; Liu et al., 2008; Hirano et al., 2011; Wei et al., 2011). We used this cassette as bait in a yeast two-hybrid screen (Y2H). After exclusion of common false positives (see Materials and methods), the most frequent hits from the Y2H were p120 catenin (four times), importin α 1a (two times), and the kinases Wee1A and B (three times and two times, respectively; Fig. S1 A). (Wee1A is maternal and Wee1B is zygotic, but both are present during embryonic epithelium formation [Okamoto et al., 2002]; hereafter we refer to them collectively as Wee1.) Wee1 drew our immediate attention, because it is a conserved, negative regulator of Cdk1 (Parker et al., 1992). Although best known for restraining entry into M phase (Russell and Nurse, 1987; Michael and Newport, 1998), recent work has implicated Wee1 in other mitotic roles, including contributing to mitotic spindle integrity in flies (Stumpff et al., 2005) and negatively regulating the metaphase-to-anaphase transition in budding yeast (Lianga et al., 2013).

**Figure 1.**
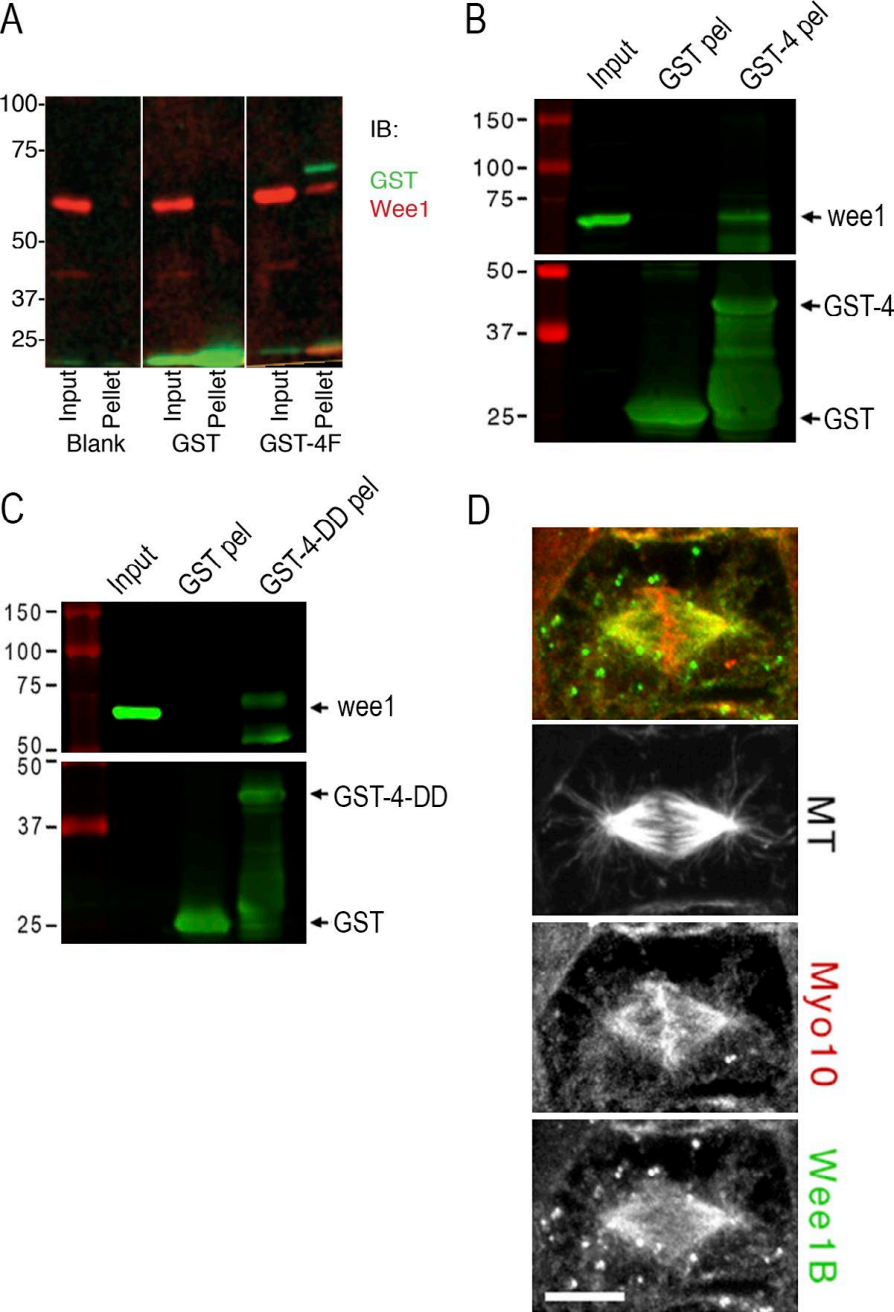
**Myo10 directly interacts with Wee1 via the MyTH4 domain. (A)** In vitro binding assay between recombinant FLAG-Wee1A and empty glutathione-sepharose beads (blank) or bead-bound GST or GST-MyTH4-FERM (GST-4F). Inputs and washed glutathione pellets were boiled in SDS sample buffer and immunoblotted for GST and Wee1 by standard methods. **(B)** Bacterial recombinant sepharose-bound GST and GST-MyTH4 (GST-4) were mixed with concentrated extracts from stage 10 *X. laevis* embryos. Washed pellets (pel) and raw extract (input) were boiled in SDS sample buffer and subjected to immunoblot analysis. Input sample represents 2% of assay input and pellets each represent half of the pull-down product. Membranes were cut at the 50-kD marker and immunoblotted for Wee1 (top) or GST (bottom). The lower GST-reactive bands in the GST-4 pel sample represent protein degradation products. **(C)** Same as B, except using recombinant GST-MyTH4-DD (GST-4-DD) protein. Numbers on the left side of the blots in A–C indicate molecular weight in kilodaltons. **(D)** Stage 10 embryos were prepermeabilized before fixation and immunostained with antibodies against α-tubulin (microtubule [MT] not in merge), Wee1B (green), and Myo10 (red). The Myo10 antibody is conjugated with Alexa Fluor 568 and was applied after the anti–rabbit secondary used to label the Wee1B antibody. Bar, 10 µm.

To confirm a direct interaction between Myo10 and Wee1, in vitro pull-down experiments were performed with purified proteins. GST-MyTH4-FERM binds FLAG-Wee1 in these assays, corroborating the Y2H results (Fig. 1 A). To confirm that Myo10 interacts with endogenous Wee1, recombinant GST-MyTH4-FERM was incubated with embryo extracts. The pellets from these pull-down experiments were positive for Wee1, as assessed with our anti–Wee1 antibody and a commercially available antibody directed against phosphorylated Wee1 (Fig. S1 B). Finally, because the isolated MyTH4 domain produces the metaphase delay, we also performed the pull-downs from embryo extracts using purified GST-tagged MyTH4 or the microtubule binding–deficient mutant MyTH4-DD. Immunoblots demonstrate that both bind to endogenous Wee1 (Fig. 1, B and C). Further, immunolocalization studies show that Wee1 and Myo10 colocalize in a poleward region of the spindle in epithelial cells (Fig. 1 D). The Myo10 localization results are consistent with previous studies of Myo10 (Woolner et al., 2008; Sandquist et al., 2016), and the spindle localization of Wee1 was confirmed with a commercial Wee1 antibody (Fig. S2). Wee1 also appears to localize to puncta that distribute throughout the cytoplasm and often colocalize with astral microtubules. These puncta are reminiscent of the cortical signaling nodes described in yeast that influence mitotic entry and contain Wee1 (Moseley et al., 2009; Deng and Moseley, 2013). Collectively, the results show that Wee1 and Myo10 interact and indicate that the MyTH4 domain alone is sufficient to bind Wee1.

### Wee1 inhibition accelerates the metaphase-to-anaphase transition

**Figure 2.**
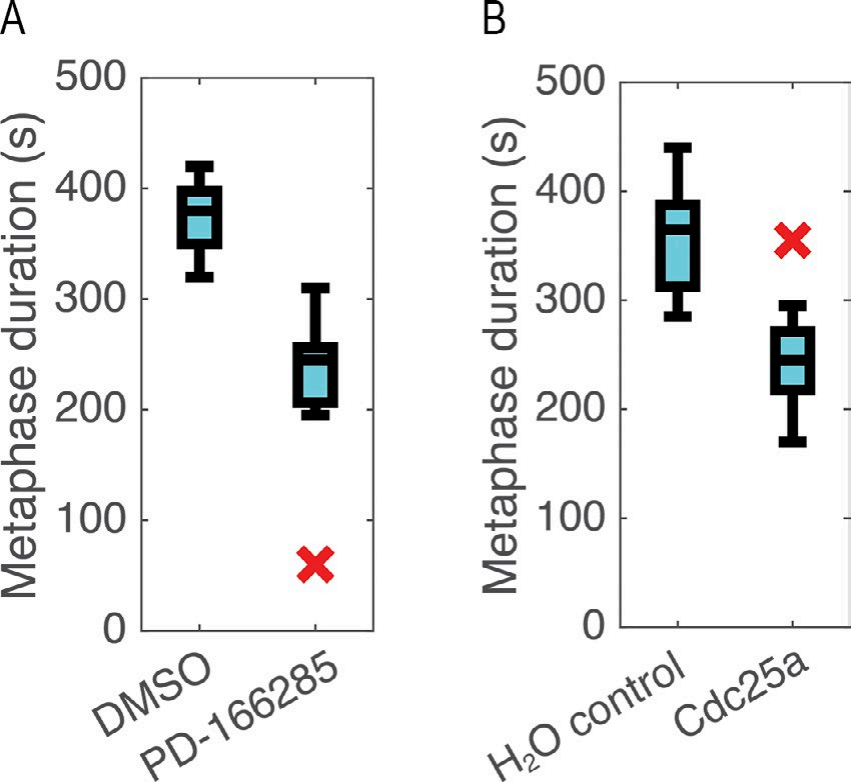
**Increasing Cdk1 activity shortens metaphase. (A)** Boxplot (median ± quartiles, X indicates outlier) of metaphase duration in DMSO control or PD-166285–treated cells reveals significant decrease with Wee1 inhibition (P = 0.005, *t* test, *n* = 13 control, 7 treated). **(B)** Boxplot of metaphase duration in cells with or without exogenous Cdc25a demonstrates a significant decrease in metaphase duration with Cdc25a overexpression (P = 0.0008, *t* test, *n* = 7 control, 15 cdc25a).

Because expression of either the isolated MyTH4 or MyTH4-DD is sufficient to produce a profound metaphase delay in *Xenopus* embryonic epithelia (Sandquist et al., 2016) the aforementioned results are consistent with the hypothesis that in vivo, the Wee1–Myo10 interaction is needed for a timely metaphase-to-anaphase transition. If this hypothesis is correct, then it would be predicted that experimental Wee1 inhibition would shorten the amount of time cells spend between metaphase and anaphase. To test this prediction, *Xenopus* embryos expressing eGFP-α tubulin and mCherry-Histone H2B were imaged after microinjection with the Wee1 inhibitor PD166285 (previously demonstrated to work in *Xenopus* embryos; Chang and Ferrell, 2013) or, as a control, vehicle (DMSO), and the time from metaphase to anaphase was measured. Consistent with the hypothesis, PD166285 significantly reduced the duration of metaphase (from ∼380 to ∼220 s; Fig. 2 A; P = 0.005). As an independent test of the hypothesis, we sought to mimic the effects of Wee1 inhibition by overexpression of Cdc25. That is, Wee1 restrains both M phase onset and the metaphase-to-anaphase transition by inhibitory phosphorylation of Cdk1 on T14 and Y15 (Gould and Nurse, 1989; Lianga et al., 2013), whereas Cdc25 promotes Cdk1 by dephosphorylation of these residues (Kumagai and Dunphy, 1991). Cdc25 overexpression closely mimicked the effects of the Wee1 inhibitor, shortening metaphase duration from ∼370 s to ∼240 s (Fig. 2 B; P = 0.0008). Thus, as in budding yeast (Lianga et al., 2013), in *Xenopus* embryonic epithelial cells, Wee1 restrains the transition from metaphase to anaphase.

### Cdk1 localizes to cell–cell junctions

**Figure 3.**
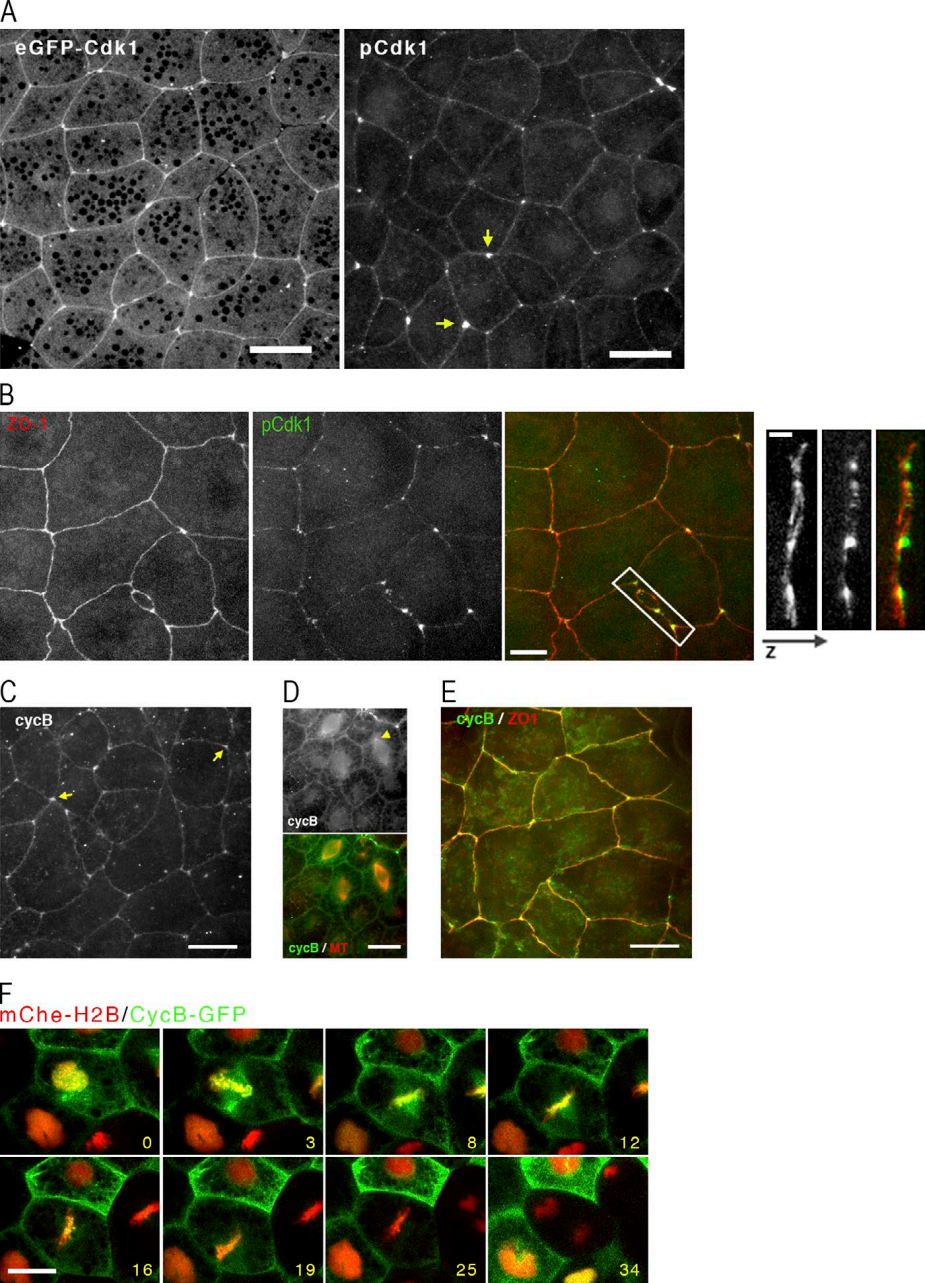
**Cdk1 is enriched at cell junctions. (A)** Left: Image from a live stage 10 embryo expressing eGFP-Cdk1. Image shown is z-projection of apical region showing the native GFP signal along junctions, particularly tricell junctions. Bar, 20 µm. Right: Stage 10 embryo fixed and immunostained with antibody specific for Y15 phosphorylated Cdk1 (pCdk1). Bar, 20 µm. Arrows show tricell junctions. **(B)** Left: Stage 10 control embryos immunostained with ZO-1 (red) and Cdk1–phosphorylated tyrosine 15 (green). Bar, 10 µm. Right: A 3D projection of the boxed region, rotated 90 degrees, with the apical surface to the right. Bar, 2.5 µm. **(C)** Apical projections of stage 10 embryo fixed in methanol/DMSO and immunostained with antibodies for cyclin B. Arrows show tricell junctions. Bar, 20 µm. **(D)** Midcell projections of stage 10 embryo fixed in formaldehyde and immunostained with antibodies for cyclin B (green) and microtubules (red). Arrowhead shows poleward spindle staining of cyclin B. Bar, 20 µm. **(E)** Apical projection of stage 10 embryo fixed in methanol/DMSO and immunostained with antibodies for cyclin B (green) and ZO-1 (red). Bar, 20 µm. **(F)** Series of still images from a movie of stage 10 embryos expressing mChe–histone H2B (red) and cyclin B–GFP (green). Time stamps indicate minutes after first frame. Bar, 20 µm. See also Video 1.

Because Wee1 exerts its effects via Cdk1 and because proper spindle positioning is ultimately a spatial problem, we next sought to characterize the distribution of Cdk1 and its binding partner, cyclin B, because to the best of our knowledge, neither of these proteins have been localized in intact epithelia before. Surprisingly, eGFP-Cdk1 concentrated at cell–cell junctions, with a particular concentration at tricell junctions (i.e., junctions where three cells come together; Fig. 3 A, left). The tricell localization was especially provocative in that spindles orient toward tricell junctions in *Drosophila melanogaster* epithelia (Bosveld et al., 2016) and the most obvious cortical landmark near “on target” touches by *Xenopus* epithelial spindles are tricell junctions (Larson and Bement, 2017). We therefore sought to independently confirm this result by immunolabeling embryos with an antibody directed against Cdk1 phosphorylated on Y15 (pCdk1), the target site for Wee1 phosphorylation. Consistent with eGFP-Cdk1 localization in live cells, anti–pCdk1 labeled cell–cell junctions with a clear enrichment at tricell junctions (Fig. 3 A, right). To compare the localization of Cdk1 to a known junctional protein, samples were double labeled for pCdk1 and the tight junction component ZO-1 (Stevenson et al., 1986; Higashi et al., 2016). pCdk1 localization paralleled that of ZO-1 along the cell–cell junctions but was found in z-projections to be concentrated just above (i.e., just apical) to the ZO-1 (Fig. 3 B). Importantly, endogenous cyclin B demonstrates a similar pattern of junctional staining (Fig. 3 C, arrows) and colocalization with ZO-1 (Fig. 3 E) in apical projections of epithelial cells, whereas midcell projections show nuclear signal and colocalization with the spindle (Fig. 3 D). eGFP-Cdk1 shows similar spindle localization as endogenous cyclin B (unpublished data). Thus, pools of cyclin B–Cdk1 localize to the spindle (consistent with prior work; Clute and Pines, 1999) and to an apical junctional compartment, with particular enrichment in tricell junctions. Further, the possibility of dynamic exchange between these pools is suggested by movies of GFP-cyclin B (Fig. 3 F and Video 1).

### MyTH4 expression increases Wee1 activity and delays metaphase in a Wee1-dependent manner

**Figure 4.**
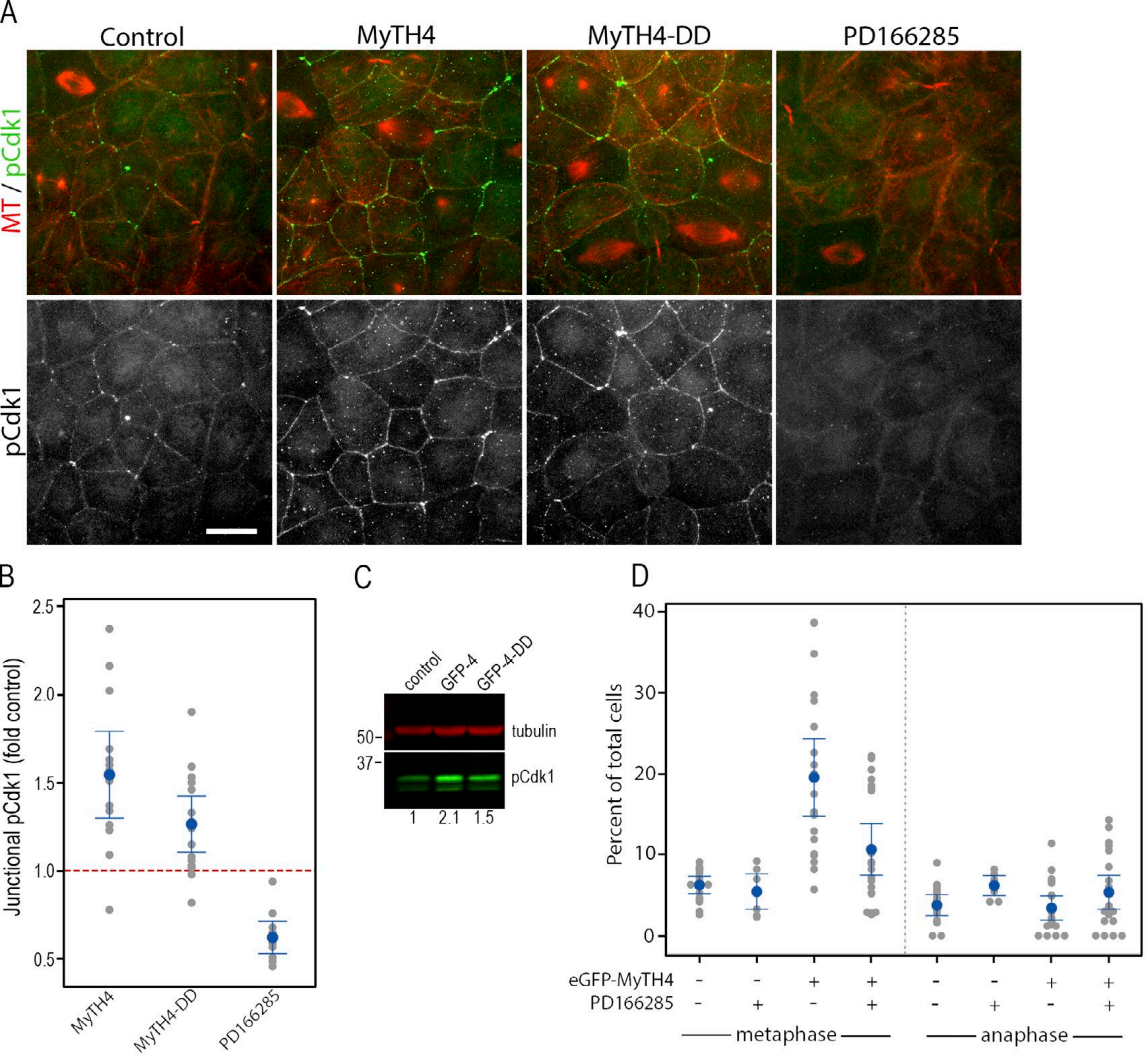
**MyTH4 domain expression increases junctional pCdk1 staining. (A)** Uninjected control stage 10 embryos, or those expressing eGFP-MyTH4 or eGFP-MyTH4-DD or treated with 25 µM PD166285 for 30 min, were fixed and immunostained with antibodies against α-tubulin (MT, red) and pCdk1 (green). Bar, 20 µm. **(B)** Quantification of junctional pCdk1 signal in uninjected control embryos, or those expressing MyTH4 or MyTH4-DD or incubated for 30 min in 25 µM PD166285. y axis represents fold change in junctional pCdk1 relative to controls within the same experiment. The red line represents the mean control pCdk1 at junctions, defined as 1. A gray dot represents a single embryo, the blue dot the mean and the blue bars the 95% confidence interval. *n* = 10–15. All are significantly different from control (from left to right: P = 0.0004; P =0.0001; P =0.003). **(C)** Embryos were microinjected at the two-cell stage with mRNA encoding either GFP-MyTH4 (GFP-4) or GFP-MyTH4-DD (GFP-4-DD). At stage 10, whole-cell extracts were prepared and immunoblotted for α-tubulin (red) and pCdk1 (green). The numbers at the bottom of the blot indicate relative levels of pCdk1 in this image. Numbers on the left side indicate molecular weight in kilodaltons. **(D)** Stage 10 control or eGFP-MyTH4-expressing embryos were incubated for 0 or 30 min in 25 µM PD166285. The embryos were then fixed and stained for microtubules (anti–α-tubulin) and DNA (DAPI or TO-PRO3). The percentage of metaphase and anaphase cells were counted for each field of epithelium taken at 40×. In the plot, a gray dot represents a single embryo, the blue dot the mean, and the blue bars the 95% confidence interval. *n* = 10–20 embryos from four different experiments.

The Wee1–MyTH4 interaction, the metaphase acceleration produced by Wee1 inhibition, and the previously demonstrated MyTH4-induced metaphase delay prompt a hypothesis in which MyTH4 expression causes metaphase arrest by increasing Wee1 levels, Wee1 activity, or both. To test this hypothesis, Wee1 activity was first monitored by comparing junctional pCdk1 levels between controls and embryos expressing MyTH4 or MyTH4-DD (Fig. 4 A). The fidelity of this approach was tested by quantifying junctional pCdk1 after treatment with the Wee1 inhibitor, which significantly reduced junctional pCdk1 localization (Fig. 4, A and B), as did dominant-negative expression of a kinase-dead mutant of Wee1 (Fig. S3 A). Both inspection (Fig. 4 A) and quantification (Fig. 4 B) show that expression of either MyTH4 or MyTH4-DD increases junctional pCdk1 relative to controls (P = 0.001 and P = 0.004, respectively). Intriguingly, quantification of individual control cells at different cell cycle stages revealed a slight decrease in junctional pCdk1 in metaphase and anaphase cells relative to interphase cells (Fig. S3).

As a second test of the hypothesis, pCdk1 abundance was assessed by immunoblotting of controls or samples expressing MyTH4 or MyTH4-DD. Both MyTH4 and MyTH4-DD elevated pCdk1 levels (Fig. 4 C; mean ± standard error fold increases in pCdk1 induced by MyTH4 or MyTH4-DD were 1.47 ± 0.28 [*n* = 4, P = 0.19] and 1.35 ± 0.15 [*n* = 5, P = 0.07]).

As a third test of the hypothesis, we determined whether Wee1 inhibition relieved the metaphase delay imposed by MyTH4 expression. Accordingly, control embryos or embryos expressing GFP-MyTH4 were fixed and stained after DMSO or PD166285 treatment and scored for the number of cells with metaphase or anaphase spindles. Consistent with the results above and previous studies (Sandquist et al., 2016), GFP-MyTH4 significantly elevated the number of metaphase spindles in the embryonic epithelia (Fig. 4 D; P = 0.001 vs. uninjected + DMSO, ANOVA with Tukey’s). The combination of GFP-MyTH4 and PD166285, in contrast, was not significantly different from controls (P = 0.30 vs. uninjected + DMSO), although the mitotic index of GFP-MyTH4 + PD166285–treated cells was significantly different from GFP-MyTH4 alone (P = 0.002). Collectively, the results indicate that the MyTH4-mediated metaphase arrest does indeed result from elevation of Wee1 activity. The molecular basis for increased Wee1 activity is unclear; although it could result from changes in Wee1 protein levels or localization, preliminary experiments indicate that Myo10 is not broadly controlling Wee1 protein levels or subcellular distribution (unpublished data). This raises the intriguing possibility that Myo10 might directly inhibit Wee1 kinase activity, a possibility that could be explored in future experiments.

### Links among Myo10, Wee1, spindle motility, and anaphase onset

**Figure 5.**
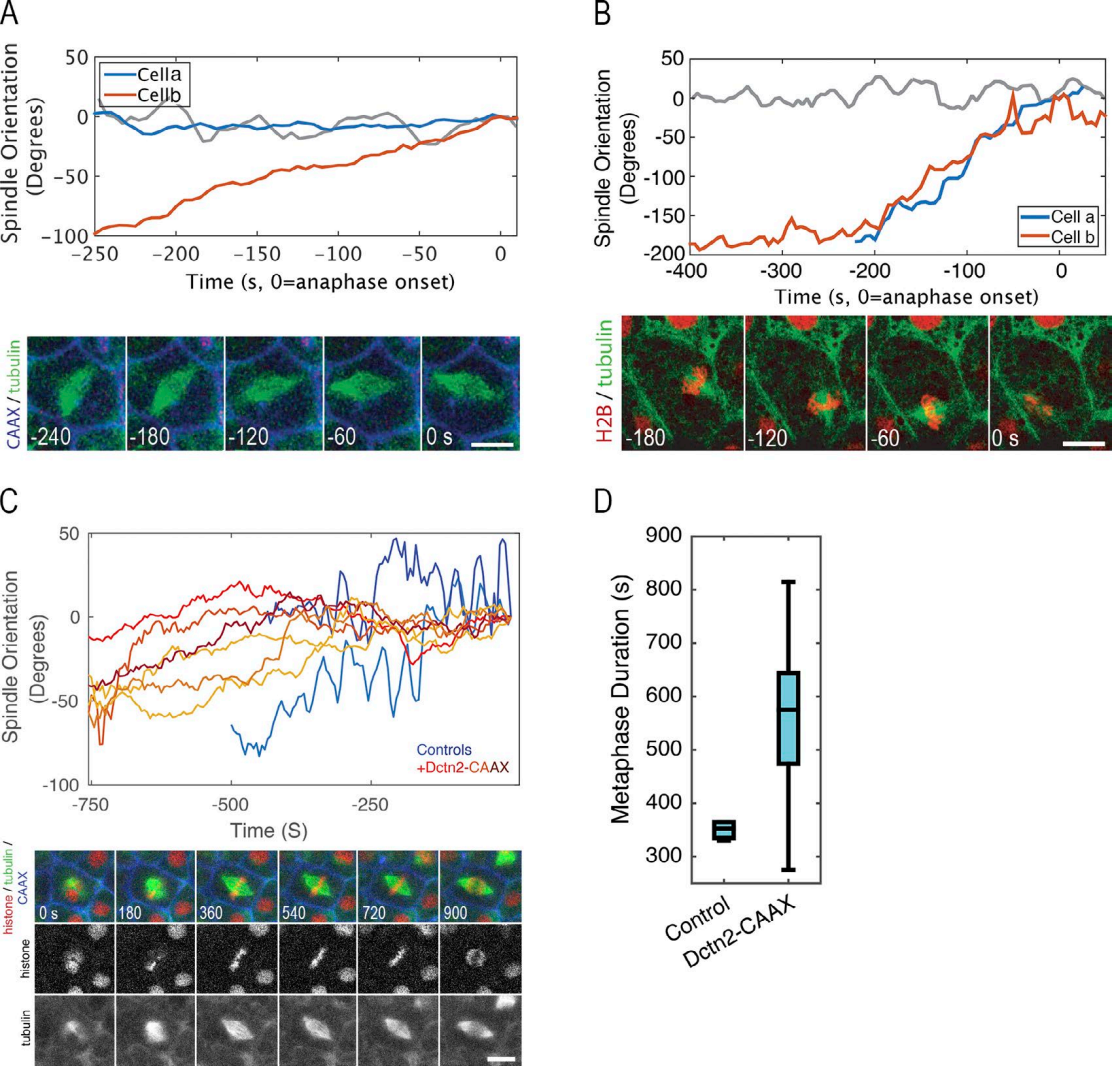
**Wee1 inhibition rescues MyTH4-induced anaphase delay and reduces spindle oscillations. (A)** Manually measured orientation of the mitotic spindle versus spindle axis at anaphase onset in cells expressing eGFP-tubulin and mTagBFP-CAAX in the presence of Myo10 morpholino (red and blue traces) or control (gray). Image shows time series of a Myo10-depleted cell expressing BFP-CAAX (blue) and eGFP-α-tubulin (green) and corresponds to the red trace. Time stamps indicate seconds before anaphase (anaphase = 0 s). Bar, 10 µm. **(B)** Same as A, except cells were treated with PD166285 (red and blue traces) or control (gray). Image shows time series of a PD166285-treated cell expressing mCherry–histone H2B (red) and eGFP–α-tubulin (green) and corresponds to the red trace. **(C)** Spindle orientation, as a function of spindle orientation at anaphase onset, as determined by the Spindlometer in control (two blue traces) and dynamitin2-CAAX-expressing (six yellow-red traces) embryos. Image shows time series of cells expressing eGFP-tubulin (green), mChe–histone H2B (red), mTagBFP-CAAX (blue), and dynamitin2-CAAX (unlabeled). Bar, 10 μm. **(D)** Manually measured metaphase duration in cells prepared as in D (control *n* = 6, Dctn2 *n* = 13, P = 0.0014).

Finally, because of the correlation between spindle motility and anaphase onset (see Introduction; Larson and Bement, 2017), we assessed potential consequences of manipulation of Myo10 levels or Wee1 activity on spindle dynamics, as well as potential consequences of spindle motility on anaphase onset. To accomplish the first of these, spindle dynamics were monitored after Myo10 depletion using a previously characterized Myo10 morpholino (Woolner et al., 2008) or after treatment with PD166285 to inhibit Wee1. Analysis of spindle dynamics revealed that Myo10 depletion has no effect on the rotational dynamics comprising the first part of the spindle dance but strongly suppresses the oscillatory cortical contacts (Fig. 5 A). Similarly, PD166285 has no obvious effect on spindle rotation but reduces oscillatory contacts (Fig. 5 B). Significantly, PD166285 treatment also occasionally resulted in anaphase onset when spindles were still asymmetrically positioned in the x–y plane (Fig. 5 B), a behavior not seen in control samples (not depicted; Larson and Bement, 2017).

The above data suggest a model in which spindle translocation to the cortex is responsible for initiating the metaphase-to-anaphase transition. Such a model predicts that suppression of spindle dynamics independently of manipulations of Wee1 or Myo10 would slow the metaphase anaphase transition. Because dynein is important for spindle dynamics in a variety of systems (Gönczy, 2008; Kotak et al., 2012; Kiyomitsu and Cheeseman, 2013), we used a novel strategy to inhibit dynein function: expression of plasma membrane–targeted dynamitin. Dynamitin is a component of the dynactin complex, and its overexpression is well established to disrupt dynein function (Burkhardt et al., 1997). However, inhibition of cytoplasmic dynein function disrupts spindle architecture by inhibiting minus end-directed transport of proteins involved in spindle pole organization (Merdes et al., 2000). We expressed a cysteine aliphatic aliphatic variable (CAAX)–modified dynamitin, which limits the localization of dynamitin to the plasma membrane (Hancock et al., 1989; Larson and Bement, 2017). This manipulation permitted formation of spindles with normal morphology while severely curtailing spindle motility. Quantification of spindle dynamics using the Spindlometer (Larson and Bement, 2017) showed that oscillatory cortical contacts by the spindle were eliminated by this manipulation (Fig. 5 C). As predicted by the model, this elimination was accompanied by a significant increase in metaphase duration (Fig. 5 D).

Our results show that, as in budding yeast (Lianga et al., 2013), Wee1 restrains the metaphase-to-anaphase transition in an intact vertebrate epithelium, presumably because of its phosphorylation of Cdk1, which is unexpectedly concentrated in a novel pool just apical to the tight junctions. The data also show that Wee1 binds to the MyTH4 domain of Myo10, that expression of isolated MyTH4 or the MyTH4DD mutant causes metaphase arrest by reducing Cdk1 activity, and that Wee1 activity and Myo10 are both required for the oscillatory spindle movements that are normally correlated with anaphase onset. Finally, the results show that independent (of Myo10 or Wee1 manipulation) suppression of the oscillatory spindle movements delays the metaphase-anaphase transition.

To the best of our knowledge, this represents the first demonstration of a myosin motor interacting with a basic cell cycle regulator, the first demonstration of a junctional pool of cyclin B–Cdk1, and the first demonstration of a role for Wee1 in the metaphase-to-anaphase transition in vertebrate cells. More importantly, however, the results indicate that timely anaphase onset in a vertebrate epithelium is normally entrained to proper spindle positioning by a mechanism that includes Wee1–Myo10 binding and spindle dynamics. Exactly how the spindle dynamics are linked to anaphase onset is not clear, but one plausible hypothesis emerges from the striking distribution of pCdk1, in particular the concentration of pCdk1 at tricell junctions, which are viewed as orientation landmarks for mitotic cells (Bosveld et al., 2016), and the relative decrease in junctional pCdk1 in mitotic cells. Similar to what has been proposed in yeast, we suggest that in early mitosis a low level of Wee1 activity keeps cyclin B–Cdk1 at submaximal levels (Lianga et al., 2013). Our previous and current results demonstrated that Myo10 is required for spindle oscillations, and our current results demonstrate that Myo10 restrains Wee1 activity. We therefore propose that as the cell approaches metaphase, Myo10, through its dual abilities to promote spindle oscillations and inhibit Wee1 activity, serves as a physical link between spindle movement and cell cycle regulatory systems. The oscillatory movements of the spindle, by bringing the spindles into close proximity to the junctions, would increase the potential for exchange between spindle and junctional proteins. In particular, active cyclin B–Cdk1 may be delivered from the spindle to the tricell junctions. Such exchange is supported by the dynamic behavior of GFP–cyclin B observed in movies of dividing cells (Video 1) and would be expected to result in an incremental increase in cyclin B–Cdk1 activity at junctions via well-described feed-forward activation mechanisms. After several such contacts, Cdk1 activity would pass the threshold needed to trigger the positive feedback loops that initiate the metaphase-to-anaphase transition (King et al., 1995; Patra and Dunphy, 1998; Rudner and Murray, 2000; Lianga et al., 2013). This model provides a mechanism for an increase in cyclin B–Cdk1 activity at the cortex coincident with a locale decrease at the spindle, such as suggested by the reported decrease in Cdk1-mediated phosphorylation of spindle-associated nuclear and mitotic apparatus at that time (Kotak et al., 2013). This hypothesis of spindle dynamics–mediated spatiotemporal regulation of Cdk1 is testable, although rigorous testing will require the development of a Cdk1 activity reporter with much higher spatiotemporal resolution than those currently available.

## Materials and methods

### DNA constructs, mRNA synthesis, and morpholino

Unless otherwise noted, the DNA encoding proteins expressed in *X. laevis* embryos were maintained in custom-made vectors based on the pCS2+ backbone (Sokac et al., 2003). Previously described protein probes include eGFP–α-tubulin (human), mChe–α-tubulin, mChe–histone H2B (human), mTagBFP-CAAX (Larson and Bement, 2017), eGFP-MyTH4 (*X. laevis*), and eGFP-MyTH4-DD (R1638D and R1641D; Sandquist et al., 2016). pCMX–cyclin B–GFP was a gift from C. Rieder (Wadsworth Center, New York State Department of Health, Albany, NY). *X. laevis* Wee1A (clone 6864894), Wee1B (clone 7010691), Cdc2 (clone 5542421), Cdc25a (clone 3378114), and Dctn2 (clone 7012124) were obtained from Open Biosystems (GE Dharmacon). The coding DNA sequences for Wee1A, Wee1B, and Cdc2 were PCR amplified to remove the start codon and add restriction enzyme sites for cloning into pDONR221 Gateway vector (Thermo Fisher Scientific) and then recombined into a house-generated pCS2-eGFP-DEST vector. Kinase-deficient (KD) Wee1B is a K270R substitution (Leise and Mueller, 2002) generated by sited-directed mutagenesis using the following primers: 5′-GCATCTACGCCATAAGGCGATCGAAGAAGCC-3′ and 5′-GGCTTCTTCGATCGCCTTATGGCGTAGATGC-3′ (Integrated DNA Technologies). The coding DNA sequences for Cdc25a and Dctn2 were amplified by PCR and cloned into pCS2 or pCS2-CAAX, respectively, by restriction enzyme digest and ligation (Dctn2-CAAX construct produced by D. Funk, University of Wisconsin-Madison, Madison, WI). For leucine-glycine-asparagine (GPSM2), a cDNA library was generated from *X. laevis* oocyte mRNA with the iScript cDNA Synthesis kit (Bio-Rad). The leucine-glycine-asparagine coding sequence was amplified from the cDNA library with primers 5′-ATGTCGATGGAGGGGACTGAAGCATTG-3′ and 5′-CTAATTAGAGTTTGGTCTTTCTAAGAGGGAGG-3′. The cDNA sequence was then amplified by PCR with extended primers containing upstream BspEI and downstream XbaI sites, and cloned into pCS2-eGFP by restriction enzyme digest and ligation. mRNA was synthesized in vitro from NsiI, NotI, or NotI-HF (New England Biolabs) linearized pCS2 vectors using the mMessage mMachine SP6 kit and vendor protocol (Ambion). Synthesized mRNAs were purified by phenol/chloroform extraction followed by isopropanol precipitation or by use of the RNEasy MinElute Cleanup kit (QIAGEN) and then resuspended to 0.5–1 g/l in nuclease-free water. The sequence of morpholino used for the depletion of Myo10 protein was 5′-TATTCCTCCATGTCTCCCTCTGCTC-3′ (Gene Tools).

### Wee1 nomenclature

*Xenopus* and humans each possess two Wee1 genes. In *Xenopus*, the embryonic form is called Wee1 or Wee1A and the somatic form is known as Wee2 or Wee1B (Okamoto et al., 2002). In humans, the nomenclature is reversed (i.e., *Xenopus* Wee1A corresponds to human Wee1B). Much of the work here is performed in early gastrula frog embryos, a time in development when both Wee1 isoforms are present at low levels (Okamoto et al., 2002).

### Y2H

A Y2H was performed using the Clontech Matchmaker Yeast Two-Hybrid System (Takara Bio). The Myo10 MyTH4-FERM bait construct was generated by PCR using *X. laevis* Myo10 as the template, with the following primers: 5′-GTCGCATATGGGGCGGAAACATTCATAC-3′ and 5′-ATTGCCCGGGTCACCTGGCCCAGTTGCT-3′ (coding amino acids 1,461–2,053). The MyTH4-FERM cassette was then ligated into the Matchmaker vector, pGBKT7, using NdeI and SmaI. The Y2H was performed according to the Protocol B-Screen by cotransformation in the Matchmaker manual, screening the bait against a cDNA library prepared from *Xenopus laevis* oocytes and early embryos. To construct the cDNA library, total RNA was extracted from oocytes and 4, 16, and 24 h postfertilization embryos (incubated at 17°C) and processed to yield poly(A)^+^ RNA with the MicroPoly(A) Purist mRNA Isolation kit (Ambion). cDNA was then prepared using a random primer, amplifying by long-distance PCR, as described in the Matchmaker manual (Takara Bio). To perform the two-hybrid screen, the yeast strain, AH109, was cotransformed with the cDNA and pGBKT7-MyTH4-FERM bait. Positive interactors were selected by blue growth on quadruple dropout/X-α-Gal medium. Prey plasmid DNA was extracted using a standard Hoffman–Winston plasmid rescue protocol (Hoffman and Winston, 1987), transformed into *E. coli* by electroporation and sequenced.

### Recombinant protein production

Bacterial expression vectors for MyTH4 and MyTH4-DD were made by restriction enzyme digestion from the pCS2-eGFP-MyTH4 and pCS2-eGFP-MyTH4-DD vectors described in the first paragraph of this section and ligation into pGEX-4T (GE Healthcare Lifesciences; Weber et al., 2004). For baculovirus-generated proteins, GST and GST-MyTH4-FERM (GST-4F) were cloned from respective pGEX-4T vectors into pFastBac1 (gift from K. Sonnemann, University of Wisconsin-Madison, Madison, WI). pFastBac1-FLAG-Wee1A was generated by PCR amplification of Wee1A from pCS2-eGFP-Wee1A using extended primers containing forward SalI and FLAG sequences and reverse Not1 sequences and then digested and ligated into pFastBac1. All GST proteins were batch purified using glutathione-sepharose 4B (GE Healthcare Lifesciences) following the supplier’s suggested protocols. In brief, bacterial or Sf9 cell pellets were solubilized in PBS supplemented with 1% TX-100 and a cocktail of protease inhibitors. Lysates were cleared by centrifugation and the cleared supernates were incubated with prewashed glutathione-sepharose beads for 1–2 h at 4°C. Glutathione pellets were washed two or three times with six bed volumes of solubilization buffer (supplemented with 50 mM NaCl and 1 mM DTT in the case of bacterial protein). For FLAG-Wee1A, Sf9 cell pellets were solubilized and cleared as above. Cleared supernate was applied to charged Anti-Flag M2 agarose (Sigma-Aldrich) column and recirculated 5 times. The column was rinsed with 20 column volumes of PBS containing PMSF and benzamidine and then eluted in 1 M arginine at pH 4.4 into neutralizing buffer. The resultant fractions were pooled and spin concentrated and then rinsed and resuspended with cold PBS containing PMSF, benzamidine, and E-64.

### Binding assays

In vitro binding assays with purified recombinant proteins were performed by mixing 50 µl of the sepharose-bound GST, GST-4F (∼50 ng), or similarly prepared empty glutathione sepharose with 1 ml solubilization buffer and 5 µl FLAG-Wee1A (∼250 ng). Samples were incubated with mixing for 1 h at 4°C, and the pellets were washed in the same manner as batch purification of GST proteins. Bound proteins were eluted by boiling the SDS sample buffer before separation by SDS-PAGE on 8–12% gradient gels and Western blot analysis. For extract pull-down assays, recombinant GST, GST-MyTH4, or GST-MyTH4-DD proteins were purified from bacteria. Approximately 1.5–2 µg of sepharose-bound protein was added to 100 µl of concentrated embryo extract (see later in this paragraph). This mixture was supplemented with PBS, protease inhibitors, TX-100 (0.1%), and 1 mM DTT to a final volume of 150 µl and incubated with rotation for 4 h at 4°C. Sepharose pellets were washed three times in 150 µl PBS supplemented with 0.1% TX-100, 50 mM NaCl, and 1 mM DTT. Bound proteins were eluted by boiling the SDS sample buffer before Western blot analysis. Concentrated embryo extracts were collected by first washing stage 10 embryos in 0.1× Modified Marc’s Ringers (MMR) containing protease inhibitors (∼0.5 ml buffer per milliliter of embryos). Washed embryos were gently packed by centrifugation with a hand-operated clinical centrifuge and as much buffer as possible was removed. Embryos were then crushed by centrifugation for 10 min at 4°C at 15,000 rpm in a SW50.1 swinging bucket rotor. The straw-colored cytoplasm was collected through the side of the tube with a 27G needle and syringe and filtered through a prewet (0.1× MMR) 0.22-µm syringe filter to remove debris.

### Embryo preparation and microinjection

Female *X. laevis* (Nasco) were induced to ovulate by injection of 800 U human chorionic gonadotropin (MP Biomedicals) into the dorsal lymph sac 18–20 h before egg collection. Eggs laid into 1× MMR (100 mM NaCl, 2 mM KCl, 2 mM CaCl_2_, 1 mM MgCl_2_, and 5 mM Hepes, pH 7.4) were fertilized within 1 h of laying. To fertilize, a macerated portion of testes was mixed with eggs in 1× MMR by gentle swirling and incubated for 1–3 min at room temperature. The mixture was diluted 10-fold with deionized water and incubated for an additional 25 min at room temperature. Fertilized eggs were dejellied in 2% cysteine (in 0.1× MMR, pH 7.8) and then washed three times in 1× MMR and three times in 0.1× MMR. Embryos were cultured in 0.1× MMR at 17°C or room temperature until microinjection. During microinjection, embryos were bathed in 5% ficoll (in 0.1× MMR). Depending on the experiment, embryos were injected at the two-cell (5 nl per cell) or four-cell (2.5 nl per cell) stages of development. Needle concentrations were as follows: Myo10 morpholino (0.25–1 mM) of eGFP-α-tubulin (0.015–0.3 g/l), mChe–histone H2B (0.008–0.015 g/l), Cdc25a (0.002–0.008 g/l), eGFP-GPSM2 (0.008 g/l), Dctn2-CAAX (0.063 g/l), mTagBFP-CAAX (0.06–0.1 g/l), eGFP-MyTH4 (0.5 g/l), eGFP-MyTH4-DD (0.5 mg/ml), eGFP-Cdc2 (0.25 g/l), cyclin B-GFP (0.6 g/l), and GFP-KD-Wee1A (0.5 mg/ml), as appropriate. Injected embryos were incubated at 17°C to approximately stage 10 (18–22 h) before fixation or imaging.

### Immunofluorescence staining

For all except ZO-1 stained samples, stage 10 embryos (developed 18–20 h at 17°C) were washed in PBS and then fixed in Superfix (100 mM KCl, 3 mM MgCl_2_, 10 mM Hepes, 150 mM sucrose, 1 mM EGTA, 3.7% paraformaldehyde, 0.1% glutaraldehyde, 0.4% NP-40, and 0.2 μM Taxol, pH 7.5) for 2–3 h at room temperature with gentle shaking. For prepermeabilization, washed embryos were incubated in buffer P (60 mM Pipes, 4 mM EGTA, 0.8 mM MgCl_2_, 18.4% [wt/vol] glycerol, and 0.1% TX-100, pH 6.8) for 1–3 min at room temperature without shaking before fixing. Fixed embryos were washed in PBS then dehydrated with multiple washes in methanol and incubated for at least 1 h at −20°C, followed by rehydration in an escalating series of PBS/methanol washes. Rehydrated embryos were bisected equatorially and then quenched in 100 mM sodium borohydride in PBS for 2–3 h at room temperature. Quenched embryos were washed in PBS and then bleached for 30–60 min in bleaching solution (5% formamide, 1% H_2_O_2_ in 0.5 × SSC [75 mM NaCl and 7.5 mM trisodium citrate, pH 7]). Bleached embryos were washed into PBST (PBS + 0.1% Tween-20) and then blocked for at least 30 min in embryo block (5% goat serum and 5% DMSO in PBST). ZO-1 samples were prepared in the same way, except they were fixed in 4:1 methanol/DMSO and the quenching step was skipped. We note that different fixatives differentially preserved various pools of localized proteins. In particular, alcohols preserve junctional cyclin B well, but not spindle-associated cyclin B, whereas the inverse is true for formaldehyde.

Blocked embryo hemisects were incubated in primary antibody in embryo block overnight at 4°C with rotation. Embryos were washed 6–8 h with rotation in PBST with four buffer exchanges. Secondary antibodies (1:500 in embryo block) were incubated overnight at 4°C with rotation and then washed as above. DNA was labeled after the postsecondary wash by incubation in either 0.1 mg/l DAPI or 0.5 µM TO-PRO3 (Molecular Probes) for 5–10 min at room temperature. For imaging, immunostained embryos were dehydrated in two or three washes in methanol then immersed in BABB (1:2 benzyl alcohol/benzyl benzoate) to clear.

Primary antibodies used were anti–α-tubulin (1:500, DM1A; Sigma-Aldrich), anti-GFP (1:500, D5.1, 2956; Cell Signaling Technology), anti–phospho-Cdc2 (Tyr15, 1:250, 9111; Cell Signaling Technology), anti–ZO-1 (1:250, 1A12; Thermo Fisher Scientific), 5–10 mg/l anti–Myo10-AF568 (see next section), and 5–20 mg/l anti-Wee1. All secondary antibodies were from Molecular Probes. The most commonly used secondary antibodies used were goat anti–mouse Alexa Fluor 488 and chicken anti–rabbit Alexa Fluor 647.

### Antibody production

Rabbit polyclonal antibodies against Wee1 were raised by Covance Research Products. The antigen was 6× His-tagged kinase domain (aa 242–518) of *X. laevis* Wee1B. The coding region was PCR amplified with extended primers that added 5′ TEV protease and NheI sites and 3′ NotI sites for cloning into pET-28a+ vector (Novagen). Antigen was purified from urea-solubilized bacterial inclusion bodies on Ni-NTA, eluted in imidazole, and dialyzed into PBS with 2 M urea. Specific IgG was affinity purified by running serum four times over a column of antigen linked to CNBr-activated sepharose 4B (GE Healthcare Life Sciences). Bound IgG was washed with 5 column volumes of 20 mM Tris, pH 7.5, and then 5 volumes of 20 mM Tris, pH 7.5, with 0.5 M NaCl. IgGs were eluted with 100 mM glycine, pH 3.2. 1-ml fractions were collected and neutralized by adding 70 µl of 1 M Tris, pH 8.8. Concentrated fractions were combined and dialyzed into PBS then supplemented with glycerol to 50% and stored at −20°C (∼0.5 g/l IgG). Rabbit polyclonal antibodies against the head domain of Myo10 (Weber et al., 2004) were produced by similar methods. Alexa Fluor 568–labeled anti-Myo10 was produced using a protein labeling kit (A10238; Molecular Probes) that links the dye to primary amines via a succinimidyl ester bond. We followed product instructions. In brief, unlabeled antibody was mixed with sodium bicarbonate to final concentration of 1.1 g/l protein and 0.1 M bicarbonate. The alkalized protein was incubated with the dye for 1 h at room temperature and then purified using the provided column. The final product was determined to contain 0.74 g/l IgG labeled at 2.5 mol of dye per mole of IgG.

### Microscopy and image analysis

All live imaging was performed on a 3-Channel point scanning confocal microscope (Prairie Technologies, now a division of Bruker) with a 60× objective (Olympus). Laser power (405, 488, and 561 nm) and photomultiplier tube voltage were adjusted to optimize image quality for each sample. Laser interlacing was used to reduce bleed-through between channels, although the 405- and 561-nm lasers were included in the same track to reduce imaging time.

Fixed images were collected on one of two microscopes. One was an IX81 microscope (Olympus) with a DSU confocal attachment (disc 2), 40× or 60× objectives, and an Orca EM camera (Hamamatsu), using Slidebook 6 software (3i). Samples were illuminated with a HBO 200 W/2 lamp (Osram), and light was collected using filter sets from Chroma Technology: FITC (49002), TRITC (49005), or Cy5 (49913). A second microscope used was a Bio-Rad MRC1024 laser scanning confocal microscope with Bio-Rad 1024 Lasersharp confocal software (Bio-Rad), using a 63× objective and illumination with 488-, 568-, or 647-nm laser lines.

Images were processed and analyzed in ImageJ or Fiji. Figures were assembled in Adobe Photoshop CS6. Metaphase and anaphase spindle counts were made manually by assessing spindle and DNA morphology. Metaphase duration was manually quantified as the time elapsed from complete formation of the metaphase plate until separation of chromosomes is apparent at anaphase onset. Spindle orientation at a given time was determined by manual measurement of spindle orientation as normalized by the spindle orientation at anaphase onset, with the exception of Fig. 5, in which the Spindlometer (Larson and Bement, 2017) was used to track spindle dynamics. Junctional pCdk1 signal was quantified manually. In brief, z-stacks of control and treated samples were collected under identical conditions. z-projections were made of the sections that included cell junctions, with the same number of optical sections used for all images within a given experiment. Junctional and nonjunctional regions were isolated in the following way: the junctions were manually traced with a line tool that was 1.75-µm thick, and the lines were colored black. The thresholding tool was used to specifically select the traced black regions. This region of interest, the junctional region, was transferred onto a copy of the original z-projection. The mean pCdk1 signal inside the junctional region was determined and corrected for background by subtracting the mean signal of the nonjunctional region. The corrected junctional signal for each individual sample image from the treated groups was normalized by dividing by the mean junctional signal from all the control images (*n* ≥ 3) within the same experiment.

### Preparation of embryo lysates and Western blotting

Stage 10 embryos were washed in PBS and then homogenized in lysis buffer (PBS, 0.5 mM PMSF, and protease inhibitor cocktail [P8340 at vendor recommendations]; Sigma-Aldrich) by repeated pipetting in a volume of 10 μl lysis buffer per embryo. Homogenates were centrifuged at 21,000 *g* for 15 min. Cleared cytoplasm (middle layer) was collected and boiled for 5 min in SDS sample buffer (final concentrations: 18 mM Tris-base, 3% glycerol, 3% SDS, 3% 2-mercaptoethanol, 3 mM DTT, and bromophenol blue, pH 6.8). Lysate equivalent to 0.5–1 embryo was loaded in a single lane and separated by standard SDS-PAGE techniques. The proteins were transferred to nitrocellulose, stained with Ponceau S, destained, and then dried. Dried membranes were either stored at 4°C or immediately rehydrated for immunoblotting. Blocked membranes (Odyssey PBS blocking buffer [927-40100]; Li-Cor) were incubated overnight at 4°C in primary antibodies in antibody buffer (blocking buffer + 0.2% Tween-20). Primary antibodies used were antitubulin (1:8,000, DM1A; Sigma-Aldrich), anti-GFP (1:1,000, 2956; Cell Signaling Technology), anti-GST (1:1,000, mouse; Sigma-Aldrich), anti-GST (1:1,000, 2625; Cell Signaling Technology), anti-mCherry (1:2,000, M11217; Invitrogen), and anti-Wee1 (1:500). Membranes were washed three times for 5 min in PBST and then incubated for 1 h at room temperature in secondary antibodies in antibody buffer (all fluorophore-conjugated secondaries, 1:10,000; Li-Cor). Membranes were washed three times for 5 min in PBST then scanned with Odyssey Infrared Imaging System (Li-Cor).

### Drug treatments

PD166285 (Tocris or Sigma-Aldrich) stocks were prepared in DMSO and frozen until use. For live imaging, stocks were diluted with nuclease-free water to 500 µM with 2% DMSO. Immediately before imaging, two 16-nl microinjections of 500 µM working solution were made directly into the blastocoel, for an approximate final PD 166285 concentration of 50 µM. Controls were injected with 2% DMSO alone. For MyTH4 rescue experiments, stock PD166285 was diluted to 25 µM in 0.1× MMR. Developing stage 10 embryos were bathed in this solution or 0.1× MMR alone for 30 min at room temperature then immediately fixed.

### Data analysis

The majority of statistical comparisons used a two-way ANOVA and Tukey’s post-hoc tests. As junctional pCdk1 signal is presented normalized to controls, which are defined as 1, statistical differences from control were made by one-sample *t* tests against a hypothesized mean of 1.0 using Minitab Express (Minitab).

### Online supplemental material

Fig. S1 A shows the top hits from a Y2H using GST-MyTH4-FERM as bait, and Fig. S1 B is a Western blot showing copurification of pWee1 with GST-MyTH4-FERM in embryo extracts. Fig. S2 (A and B) demonstrate the localization of Wee1 and pWee1 at the mitotic spindle, respectively. Fig. S3 A shows the loss of junctional pCdk1 staining upon expression of GFP-KD-Wee1 in frog epithelia, whereas Fig. S3 B is a plot demonstrating that junctional pCdk1 signal is decreased in metaphase and anaphase cells compared with interphase cells. Video 1 shows the dynamic localization of GFP–cyclin B in actively dividing frog epithelial cells and corresponds to the still images in Fig. 3 F. Video 2 demonstrates the reduced spindle dynamics in cells expressing dynamitin and corresponds to the still images in Fig. 5 C.

## Supplementary Material

Supplemental Materials

Video 1

Video 2
