# Supplementary material for: An interaction between myosin-10 and the cell cycle regulator Wee1 links spindle dynamics to mitotic progression in epithelia
Source: J Cell Biol. 2018 Mar 5;217(3):849–59. doi: 10.1083/jcb.201708072 (PMC5839792; doi:10.1083/jcb.201708072)
Supplement: Supplemental Materials [file JCB_201708072_sm.pdf]

Supplemental material

Sandquist et al., https://doi.org/10.1083/jcb.201708072

JCB

A

Hit name	# of hits	GenBank #	Function
X. laevis XNCp120.1.2. p120 isoform 1	4	AF150744.1	cell adhesion
X. laevis Wee1A kinase	3	BC081031.1	cell cycle regulatory kinase
X. laevis Wee1B kinase	2	BC082404.1	cell cycle regulatory kinase
X. laevis importin alpha 1a	2	BC097584.1	nuclear import adapter protein

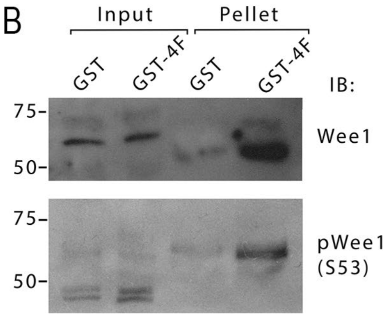

Figure S1. **Interaction between *X. laevis* Myo10 and Wee1. (A)** A Y2H using the MyTH4-FERM cassette from the tail of *X. laevis* Myo10 as bait identified Wee1 as a Myo10 binding partner. All hits identified more than once in the screen are shown. **(B)** GST-MyTH4-FERM pulls endogenous Wee1 and pWee1 out of extracts. Bacterial recombinant sepharose-bound GST and GST-MyTH4-FERM (GST-4F) were mixed with concentrated extracts from *X. laevis* embryos. Raw extract (input) and washed pellets were boiled in SDS sample buffer and subjected to immunoblot analysis. Here two identical gels were run and transferred to nitrocellulose and then immunoblotted (IB) with our Wee1 antibody (top) or a commercial antibody directed against Ser53-phosphorylated Wee1 (pWee1 S53).

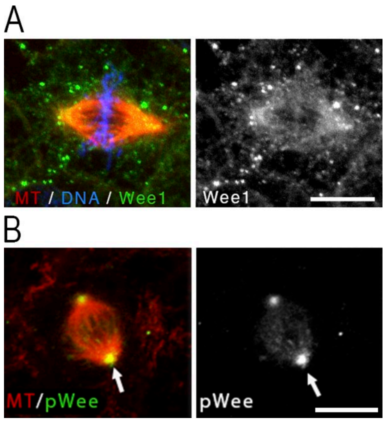

Figure S2. **Wee1 localizes to the mitotic spindle. (A)** Stage 10 embryos were prepermeabilized before fixation and immunostained with a commercial antibody against α-tubulin (microtubule [MT], red) and our Wee1 antibody (green). DNA is labeled with propidium iodide (blue). Bar, 10 µm. **(B)** Stage 10 embryos, without prepermeabilization, were immunostained for α-tubulin (MT, red) and with a commercial antibody against Ser53-phosphorylated Wee1 (pWee, green). Arrows indicate pole concentration of pWee. Other commercial antibodies against total Wee1 did not work in frog cells. The pWee antibody was used simply to verify Wee1 localization to the spindle, and we do not speculate on the relation of this phosphorylation to our model. See Watanabe et al. (2005) for a description of Ser53 pWee function. Bar, 10 µm.

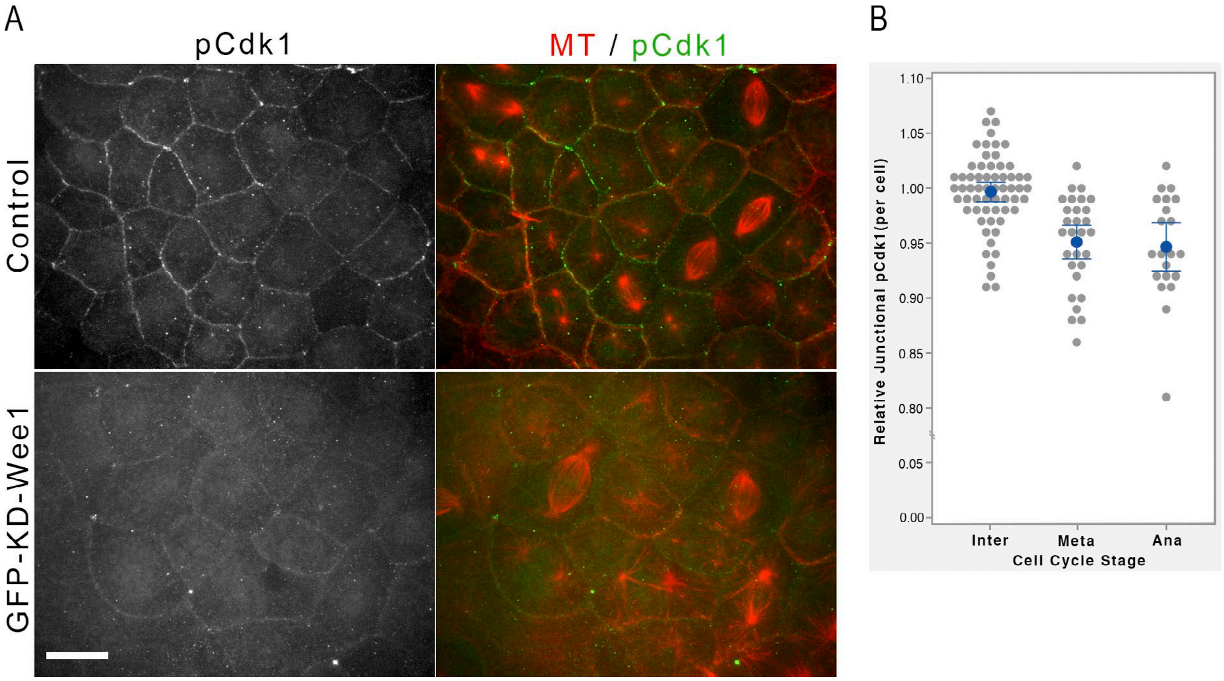

Figure S3. **Effects of kinase-dead Wee1 and cell cycle stage on junctional pCdk1 levels. (A)** Stage 10 control embryos or those expressing GFP-tagged KD Wee1A were fixed and immunostained for α-tubulin (microtubule [MT], red) and Y15-phosphorylated Cdk1 (pCdk1, green). Images shows loss of junctional pCdk1 signal in KD-Wee1A expressing cells. KD mutant was generated via a K239R substitution (Leise and Mueller, 2002). Bar, 10 µm. **(B)** Quantification of junctional pCdk1 signal in individual control cells at different cell cycle stages (ana, anaphase; inter, interphase; meta, metaphase). Y-axis represents fold change in junctional pCdk1 relative to mean interphase cell signal in the same image. A gray dot represents a single cell, the blue dot the mean, and the blue bars the 95% confidence interval. Sample size = 61 (interphase), 30 (metaphase), and 21 (anaphase). Only mitotic cells completely surrounded by interphase cells were analyzed. ANOVA and Tukey's post-hoc test showed statistically significant decreases in junctional pCdk1 for both mitotic populations compared with interphase cells ($P = 0.0001$ for both).

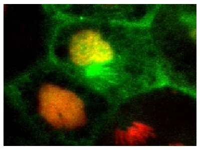

Video 1. **Stage 10 epithelial cells expressing cyclin B–GFP (green) and mCherry–histone H2B (red).** Frames represent 30-s intervals. Playback rate is six frames per second. Note the flares in cyclin B–GFP at the cortex when the spindle contacts it. Also, the spindle-associated cyclin B–GFP decreases before cytoplasmic protein, but complete cellular depletion occurs before anaphase onset.

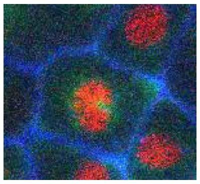

Video 2. **Spindle dynamics in cell expressing dynamitin (Fig. 5 C).** Live movie from stage 10 epithelial cells expressing eGFP–α-tubulin (green), mCherry–histone H2B (red), mTagBFP-CAAX (blue), and dynamitin2-CAAX (unlabeled in movie). Frames represent 5-s intervals. Playback rate is 24 frames per second.

References

Leise, W. III, and P.R. Mueller. 2002. Multiple Cdk1 inhibitory kinases regulate the cell cycle during development. *Dev. Biol.* 249:156–173. https://doi.org/10.1006/dbio.2002.0743

Watanabe, N., H. Arai, J. Iwasaki, M. Shiina, K. Ogata, T. Hunter, and H. Osada. 2005. Cyclin-dependent kinase (CDK) phosphorylation destabilizes somatic Wee1 via multiple pathways. *Proc. Natl. Acad. Sci. USA.* 102:11663–11668. https://doi.org/10.1073/pnas.0500410102
